# Simultaneous analysis of plasma and CSF by NMR and hierarchical models fusion

**DOI:** 10.1007/s00216-012-5871-4

**Published:** 2012-03-07

**Authors:** Agnieszka Smolinska, Joram M. Posma, Lionel Blanchet, Kirsten A. M. Ampt, Amos Attali, Tinka Tuinstra, Theo Luider, Marek Doskocz, Paul J. Michiels, Frederic C. Girard, Lutgarde M. C. Buydens, Sybren S. Wijmenga

**Affiliations:** 1 Institute for Molecules and Materials, Analytical Chemistry/ Biophysical Chemistry, Radboud University Nijmegen, Heyendaalseweg 135, 6525 AJ Nijmegen, The Netherlands; 2 Abbott Healthcare Products B.V., 1381 CP Weesp, The Netherlands; 3 Department of Neurology, Erasmus University Medical Centre, Rotterdam, 3015 CE Rotterdam, The Netherlands; 4 Spinnovation Analytical, Toernooiveld 1, 6525 ED Nijmegen, The Netherlands

**Keywords:** EAE, Multiple sclerosis, Metabolomics, Data fusion, Classification, Variable selection

## Abstract

Because cerebrospinal fluid (CSF) is the biofluid which interacts most closely with the central nervous system, it holds promise as a reporter of neurological disease, for example multiple sclerosis (MScl). To characterize the metabolomics profile of neuroinflammatory aspects of this disease we studied an animal model of MScl—experimental autoimmune/allergic encephalomyelitis (EAE). Because CSF also exchanges metabolites with blood via the blood–brain barrier, malfunctions occurring in the CNS may be reflected in the biochemical composition of blood plasma. The combination of blood plasma and CSF provides more complete information about the disease. Both biofluids can be studied by use of NMR spectroscopy. It is then necessary to perform combined analysis of the two different datasets. Mid-level data fusion was therefore applied to blood plasma and CSF datasets. First, relevant information was extracted from each biofluid dataset by use of linear support vector machine recursive feature elimination. The selected variables from each dataset were concatenated for joint analysis by partial least squares discriminant analysis (PLS-DA). The combined metabolomics information from plasma and CSF enables more efficient and reliable discrimination of the onset of EAE. Second, we introduced hierarchical models fusion, in which previously developed PLS-DA models are hierarchically combined. We show that this approach enables neuroinflamed rats (even on the day of onset) to be distinguished from either healthy or peripherally inflamed rats. Moreover, progression of EAE can be investigated because the model separates the onset and peak of the disease.

**Electronic supplementary material:**

The online version of this article (doi:10.1007/s00216-012-5871-4) contains supplementary material, which is available to authorized users.

**Figure:**
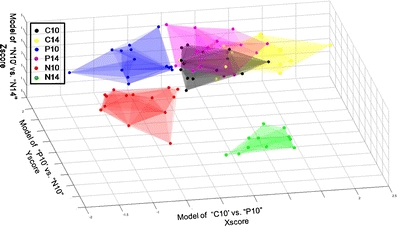
Graphical representation of Hierarchical Models Fusion applied to concatenated plasma and CSF datasets.

## Introduction

Multiple sclerosis (MScl) is an inflammatory, presumably autoimmune, disease of the central nervous system (CNS) in which the fatty myelin sheaths which surround the axons of the brain and spinal cord are damaged, leading to demyelization [1]. MScl is one of the most common neurological diseases affecting young adults and has enormous effect on the health system and economy of many countries. Although the cause of MScl is still elusive, it is believed to be a combination of genetic and environmental factors with a possible infectious origin. Signs of MScl can be observed not only in CNS but also in the peripheral nervous system (PNS) [2].

Diagnosis of MScl is still challenging, especially in its early stage. Currently, diagnosis of MScl is mostly based on clinical evidence complemented with laboratory investigations, for example presence of lesions in the brain and/or spinal cord (visualized by magnetic resonance imaging, MRI). However, lesions have been found in other neurological diseases, for example Guillain–Barré syndrome [3], and in non-neurological diseases, for example systematic vasculitis [4] or sarcoidosis [5]. Furthermore, brain lesions have been found in healthy individuals [6]. Therefore, brain lesions are not sufficiently specific for proper, early diagnosis. To improve diagnosis of MScl it is necessary to combine information from cerebrospinal fluid analysis, MRI results, and all clinical symptoms.

To fingerprint MScl at the molecular level, biological samples must be analyzed. Because CSF is the biofluid in direct contact with the brain and spinal cord, it is the most suitable choice for fingerprinting MScl. Investigation of the biochemical composition of CSF may reveal abnormal status of the brain. CSF is absorbed into the blood via a semi-permeable membrane, the blood–brain barrier (BBB). Therefore, effects of CNS diseases can potentially also be seen in the biochemical composition of blood plasma. Obviously, cross-over effects from the plasma to the CSF may also cause changes in the biochemical composition of the CSF. In MScl, the BBB is often damaged, causing “leakage” [7]. This suggests that plasma may contain predictive information about the disease. Therefore we propose to study the metabolic profiles of both CSF and plasma. These types of sample are relatively difficult to obtain from humans and interesting information is very often obscured by other factors, for example genetic, environmental, and dietetic background. Thus we opt for the possibility of using samples from designed and controlled experiments in rodents.

The animal model of MScl, the experimental autoimmune/allergic encephalomyelitis (EAE) model has become an important tool in studies of neuroinflammatory aspects of MScl [8, 9]. EAE is a cell-mediated experimental autoimmune disorder of the CNS and shares its clinical expression and pathological picture with that of MScl. EAE is used as a pre-clinical model of a single episode of MScl. Similar to MScl, in EAE a strong increase in infiltration of the BBB occurs, which leads to increased exchange between CSF and plasma.

In this study we extracted CSF and plasma samples at two time points during progression of the disease, namely at the onset and the peak; these samples were obtained from healthy, immune booster (a group of animals injected with complete Freund adjuvant emulsion, CFA), and EAE (resembling MScl) Lewis rats. The metabolic profile of the CSF and plasma was measured using the untargeted and unbiased technique of high-field 1D proton nuclear magnetic resonance (^1^H NMR). This method enables analysis of both biofluids with a very similar measurement procedure. The ^1^H NMR data of each biofluid can be analyzed separately or the two complementary NMR data sets can be combined (fused) in the analysis. In this work, CSF and blood plasma NMR spectra were used in a mid-level data fusion. The metabolite information extracted for each biofluid can be directly converted into relative concentrations for each biofluid and compared.

To obtain such information from the individual or combined data sets several analytical challenges must be solved. First, the disease must be distinguished from the healthy condition but also from other diseases, for example peripheral inflammation. This means we must construct a multi-class classifier. Second, even if well controlled the experiment still carries additional variances unrelated to the study, i.e. biological and experimental variations. Third, the number of variables recorded by NMR is much larger than the number of samples, which implies specific statistical problems. Moreover most of these variables are probably unrelated to the studied problem or are redundant. To solve these problems we propose the following architecture for the data analysis. Linear support vector machine recursive feature elimination (SVM-RFE) is used as variable-selection technique for both data sets. The selected variables are fused and analyzed using either one multi-class partial least-squares discriminant analysis (PLS2-DA) model or multiple two classes PLS-DA models, the latter using a novel approach in which a hierarchical structure enables introduction of prior knowledge. We introduce this method as hierarchical models fusion (HMF). We show that by using HMF, EAE-affected rats can be distinguished from either healthy or peripherally inflamed rats on the day of onset (when no physical symptoms of neuroinflammation are present) with 100% correct classification. In addition, the progression of EAE can be described. In summary, HMF enables simultaneous characterization of all the groups studied without applying multiclass classifier.

## Materials and methods

### Experimental design of EAE models

Experimental autoimmune/allergic encephalomyelitis (EAE) is the animal model commonly used for studying neuroinflammatory aspects of the autoimmune disease multiple sclerosis (MScl). The experimental arrangement was as previously described by Smolinska et al. and/or Hendricks [10, 11]. Here, we briefly summarize the main points. Three sets of male Lewis rats (Harlan Laboratories, the Netherlands) were inoculated on Day 0. First, a set of 30 animals was injected with guinea pig myelin basic protein (MBP), complete Freund adjuvant H37 RA (CFA, Difco Laboratories, Detroit, MI, USA) and *Mycobacterium tuberculosis* type H37RA (Difco). Another group of 30 animals was injected with CFA only. Next to these MBP and CFA challenged rats, referred to as the EAE and peripherally inflamed groups, respectively, a healthy group undergoing anaesthesia only (healthy control) was included. In each group, half of the animals were sacrificed to collect both CSF and plasma on Day 10 (onset of disease in the EAE group) and the other half on Day 14 (peak of disease in the EAE group). Typical progression of the disease is shown in Fig. S1 in the Electronic Supplementary Material, and details of the design of the EAE experiment are summarized in Table 1.

**Table 1:** Experimental design of EAE model

Group	Inflammation type	Day 10	Day 14
Healthy	None	C10	C14
		*n* = 15	*n* = 15
		*p* = 14	*p* = 14
CFA	Peripheral	P10	P14
		*n* = 15	*n* = 15
		*p* = 14	*p* = 15
CFA+MBP	Peripheral & neuroinflammation	N10	N14
		*n* = 15^a^	*n* = 15^b^
		*p* = 14	*p* = 11

“*n*” indicates the number of rats; “p” indicates the number of common samples between CSF and plasma

^a^One CSF sample was discarded because of blood contamination and one from blood plasma because of sampling

^b^Three CSF samples were discarded because of blood contamination and two from blood plasma because of sampling and preparation

### CSF and plasma sampling, sample preparation, and data acquisition

On Days 10 and 14, animals were euthanized with CO_2_/O_2_, and blood and CSF were collected. Sampling, sample preparation, and acquisition of CSF NMR spectral data were as described elsewhere [11]. Blood was sampled intravenously by use of a heparin-treated syringe. Next, every blood sample was centrifuged for 10 min at 4 °C with a relative centrifugal force of 2,000 *g* to separate the plasma. After centrifugation, samples of the supernatant were stored at −80 °C for further analysis.

For the NMR measurements, stored frozen plasma (50 μL) was left at room temperature to thaw. Next, the plasma sample was diluted with 200 μL water and the proteins were then removed by centrifugation for 30 min at 2,000 *g* (filter 10 kDa Centrisart I 13239-E; Sigma–Aldrich, St Louis, MO, USA) [12]. After protein removal, the supernatant was lyophilized. Before NMR measurements the lyophilized plasma samples were re-dissolved in 50 μL of water, after which 550 μL buffer solution was added to furnish a volume sufficient for NMR measurement. The buffer solution consisted of 2.85 mmol L^−1^ TSP-d_4_ (sodium 3-(trimethylsilyl)propionate-2,2,3,3-d_4_) (99 atom% D), 6.92 mmol L^−1^ sodium azide (NaN_3_), 42.08 mmol L^−1^ dibasic sodium phosphate dehydrate (Na_2_HPO_4_.2H_2_O), and 7.30 mmol L^−1^ HCl solvated in a H_2_O–D_2_O (99.96 atom% D) mixture (7.93:1). The final TSP concentration in each plasma sample was 2.61 mmol L^−1^.

^1^H NMR spectra of 86 plasma samples were acquired on an Avance III (Bruker BioSpin, Bruker, Billerica, MA, USA) 500-MHz system equipped 5-mm cryoprobes, CPTCI (1H-13C/15N/2H + Z-gradients) (Bruker BioSpin). Water suppression was achieved by pre-saturation. For each 1D ^1^H NMR spectrum 256 scans were accumulated with a spectral width of 10,273 Hz resulting in a total of 18 K points. The acquisition time for each scan was 3.2 s. Between scans, a 4-s relaxation delay was used. Before spectral analysis, all acquired free induction decays (FIDs) were zero-filled to 32 K data points, multiplied with a 0.3 Hz line-broadening function, Fourier transformed, manually phased, and the TSP internal reference peak was set to 0 ppm by use of ACD/SpecManager software version 12.0 [13]. All 86 rat CSF spectra were acquired and preprocessed as described elsewhere [11]. However, because of high line broadening of the internal standard (TSP) four spectra from CSF and plasma were not included in the spectral analysis. Ultimately, 82 CSF spectra and 86 plasma spectra were transferred to Matlab (version 7.9; Mathworks, Natick, MA, USA) for further analysis. Overlap between both CSF and plasma spectra was observed for 82 of these samples (Table 1).

### Preprocessing of CSF and plasma NMR spectra

The ^1^H NMR spectral data was preprocessed in Matlab; this typically involved baseline correction, alignment, binning, normalization, and scaling. For four samples (out of the 86) only plasma ^1^H NMR spectra were available. Therefore these four spectra were not used in the pre-processing and analysis process. Baseline correction of NMR spectra was performed by applying the asymmetric least-squares method [14]. Fluctuations in chemical shift were removed by applying improved parametric time warping (I-PTW) [15]. Each CSF and plasma spectrum was normalized to a total area-under-curve (AUC) of 1, to correct for potential differences in sample concentration. To reduce the high dimensionality of the data, binning was performed by means of adaptive intelligent binning [16]. This procedure led to 409 bins for CSF and 478 for plasma, which can be regarded as relative metabolites concentrations. Absolute quantification of metabolites in CSF and plasma samples was not performed and used. Data analysis was performed on binned data, i.e. on relative metabolite concentrations. The final step of preprocessing consisted of autoscaling.

### Data analysis

Explorative analysis by means of robust principal-component analysis (R-PCA) was first used to control the presence of outliers in both datasets [17]. The strategy for supervised data analysis consisted of data division into a training set (75% of samples per class) and an independent test set (25% of samples per class) by using the Duplex algorithm [18], variable selection by support vector machine recursive feature elimination (SVM-RFE) for linear kernels [19] performed on each dataset (CSF and plasma) separately, and discriminant analysis by PLS-DA [20] performed on both individual and fused datasets. For data fusion, so-called mid-level data fusion architecture was used [21]. In this approach the two data sources are first pre-processed and analyzed separately to extract relevant information; next they are fused and analyzed as a unique dataset. We used this method because it was shown to eliminate redundancy of variables. Particular steps of this type of data fusion are described in the sections “SVM-RFE” and “Classification of individual and fused plasma and CSF datasets”. In this fusion approach every data source is treated separately for pre-processing, scaling, and variable selection. Next, the optimum set of variables is combined into a single set and analyzed by PLS-DA. In the last step of data analysis the approach for cumulative fusion by means of hierarchical models fusion (HMF) was carried out. This method, proposed for the first time in this paper, is described in detail in the section “Hierarchical models fusion”. The results of this method are compared with those from PLS2-DA, a variation of PLS-DA which enables more than two groups to be analyzed simultaneously (see the section “Hierarchical models fusion”).

#### SVM-RFE

SVM-RFE was originally proposed by Guyon et al. [19] and applied to a microarray dataset in a cancer study. The method is based on the binary classification method SVM. This technique first maps objects into a feature space by use of kernel transformation and then tries to find a hyperplane which separates the data into two classes [22]. From all the separating hyperplanes, SVM looks for the one that gives the biggest separation between the borderline training samples of the two classes. The borderline training samples are called support vectors. All support vectors have an alpha value, indicating how supporting this object is for the position of the hyperplane. Non-supporting objects have an alpha value equal to 0 whereas alpha equal to 1 indicates the highest support. RFE is a backward-elimination algorithm which ranks features on the basis of the weights of linear SVM. The algorithm starts with a full training set to train a linear SVM. Next, the variables are ranked by sorting, in descending order, the square of the SVM’s weights {w_j_^2^}:

1 $$ {w_{\text{j}}}^{{2}} = {\left( {{\sum_i}_{{ = \varphi }}{\alpha_i}{y_i}{x_{{ij}}}} \right)^{{2}}} $$

where *φ* contains the indexes of support vectors, *α*_*i*_ are alpha values and *y*_*i*_ are the class labels. A variable with smallest weight *w*_j_ is then removed. Indeed, the smaller the weight of a variable, the less it contributes to the size of the margin between classes. The remaining variables are used to train another linear SVM and the entire process is repeated until all variables have been eliminated. In the work described in this paper, one variable was removed in each iteration.

We used a leave-one-out (LOO) cross-validation (CV) approach to select the optimum set of variables per data set. In this procedure one sample from the training set is left out and a variables ranking is obtained on the basis of the remaining objects. The procedure is repeated until every object is left once. The final ranking was obtained by sorting the variables on the basis of the number of times it was selected in the LOOCV procedure. The variables selected median + 1 times made up the optimum set. The complete scheme for LOOCV can be found in the Electronic supplementary material.

#### Classification of individual and fused plasma and CSF datasets

After selecting the optimum set of variables, the features of both data sets were concatenated and autoscaled. Subsequently, the variables of the fused sets were ranked by SVM-RFE. Classification of fused sets was performed by PLS-DA, a well-known method used in many omics fields [20, 23]. PLS-DA uses group information to maximize the separation between groups of observations. It is currently widely used in metabolomics because of its ability to cope with high correlations between variables. In PLS-DA a linear model is constructed in accordance with Eq. (2):

2 $$ {\mathbf{y}} = {\mathbf{Xb}} + {\mathbf{r}} $$

where **X** is a dataset matrix, **y** a vector of group memberships, **b** a vector of regression coefficients (i.e. weights of individual variable), and **r** a vector of model residuals. The regression coefficients reflect the relative importance of the variables in the PLS-DA model. PLS2-DA is a variation of PLS-DA, where the response “**y**” is not a vector but a matrix, which enables more than two groups to be analyzed simultaneously.

The optimum complexity (i.e. number of latent variables, LV) for all individual and fused models was determined by LOOCV performed on training sets. The optimum number of LV was selected on the basis of the minimum error of the root-mean-square error of cross-validation (RMSECV). For all individual and fused models the optimum model complexity was determined to be 1LV. All PLS-DA models were validated with an independent test set. A PLS-DA model is considered statistically valid if it has good prediction ability. After validation, a final model is then reconstructed using all available samples. The model can be visualized in a score plot. The importance of all variables on the predictive model can be investigated by means of the regression coefficients [24].

After the individual two-class (binary) models have been are optimized they can be used for HMF.

#### Hierarchical models fusion

In this paper, we propose a new approach, hierarchical models fusion (HMF), which uses hierarchically multiple simple two-class classification models to represent individually specific parts of the inter-class variation. This approach uses, as any supervised method, a-priori knowledge of the classes (for instance, type of inflammation) and establishes commonalities between them.

The use of simple two-class models makes the results easier to interpret. The objective of the method proposed here is to describe the relevant differences gradually instead of explaining all variation from all classes at once. This gradual process becomes possible by applying statistically optimized binary models to the data at each step and then combining the outcomes. Because it fuses the outcome of all earlier optimized models it describes and shows all the relevant differences in the data. By using this approach it is possible to visualize separation between studied classes and the relationship between objects without applying multiclass classification models (for example PLS2-DA or linear discriminant analysis, LDA).

**Fig. 1:**
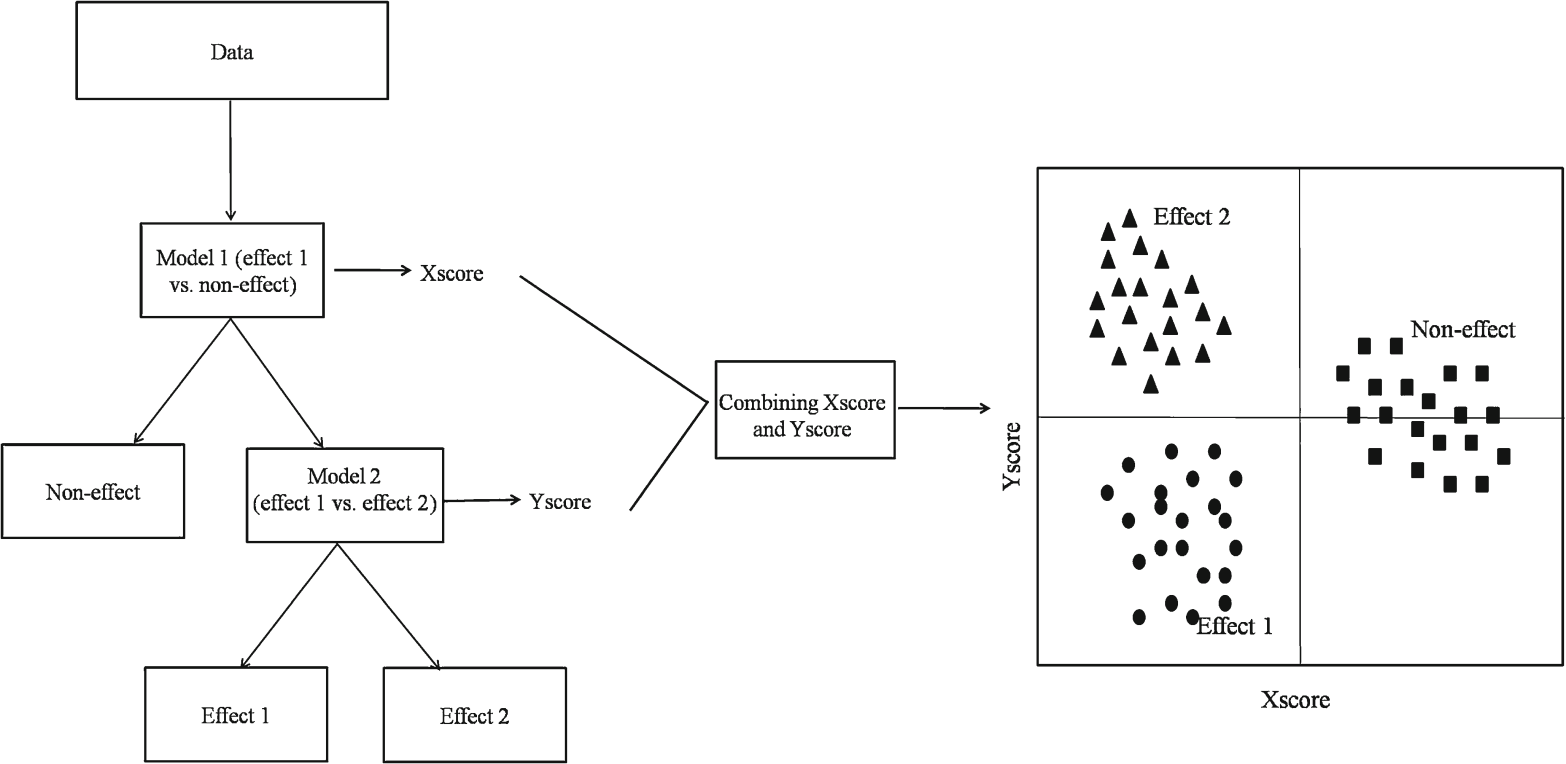
A graphical representation of hierarchical models fusion

To demonstrate the HMF approach, let us consider a dataset with three classes: non-effect (i.e. healthy), effect 1 (e.g. peripheral inflammation), and effect 2 (e.g. neuroinflammation). First, individual binary PLS-DA models of interest have to be optimized (i.e. models 1 and 2 from Fig. 1). A graphical representation of HMF is shown in Fig. 1. These models can then be hierarchically applied to the data in accordance with a-priori knowledge (here experimental design). For example having data containing three classes, i.e. effect 1, effect 2, and non-effect, it is possible to use HMF to separate all three classes. In the first step, model 1 (effect 1 versus non-effect) is used to obtain a new score for all samples in data matrix **X**. This new score (here called Xscore) separates non-effect objects from objects belonging to group effect 1 and group effect 2. In the next step, another model (model 2: effect 1 versus effect 2) can be applied to matrix **X** to assess and distinguish these two effects. In that way a second score is obtained for all samples in data matrix **X** (here called Yscore). At each step, a new score is obtained by multiplying data matrix **X** with PLS-DA weights. These two new scores (Xscore and Yscore) can then be combined and used to visualize the relationship between studied groups. When the new scores are orthogonal they can be represented as usual (i.e. with perpendicular axis).

Because HMF is based on a hierarchical structure, the complexity of the studied problem is reduced by describing every difference on a different level (i.e. step). It decomposes the difficulty of multi-class separation into simpler, solvable two-class problems. Indeed, the representation of HMF as a decision tree (Fig. 1) is similar to the classification and regression trees (CART) [25]. However, in HMF at each step (node) not a single variable but a PLS-DA model is used to separate objects. To represent the usefulness of HMF for analyzing multiple classes, simulated data were created. The results are shown in the Electronic supplementary material.

Obviously, the presented method can be used not just for visualization of relationships between samples but also for prediction of new samples (e.g. from an extra experiment). Moreover, information about variables significant for discrimination is associated with PLS-DA weights. Therefore biological interpretation is feasible also.

Any results obtained by predictive methods must be validated before drawing any conclusions. In HMF the validation is twofold. First, all the individual PLS-DA models are validated using independent test sets. Second, the complete HMF structure is also validated. Moreover to reduce the possibility of random classification we performed a permutation test for HMF.

### Metabolite identification

Metabolite identification of the most relevant set of variables was carried out by using the 800 MHz library (for CSF) and the 500 MHz library (for plasma) of metabolite NMR spectra from the Chenomx NMR Suite 7.0 (Chenomx, Edmonton (AD), Canada). The libraries of metabolite spectra were obtained on the basis of a database of pure compound spectra acquired by use of a particular pulse sequence and acquisition conditions, namely, the NOESY-presaturation pulse sequence with 4 s acquisition time and 1 s recycle delay [26]. The Chenomx NMR Suite software fits the spectral signatures (singlets, doublets, triplets etc), i.e. the peak shapes, of a compound from an internal database of reference spectra to the experimental NMR spectrum.

## Results

### Explorative analysis of the CSF and plasma datasets

**Fig. 2:**
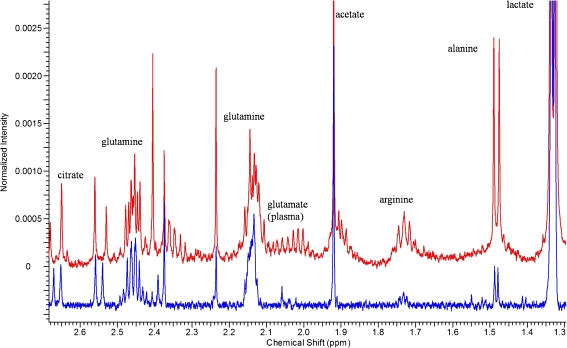
Section of the 800 MHz ^1^H NMR spectrum of CSF (*blue*) and the ^1^H 500 MHz NMR spectrum of plasma (*red*)

The 82 sets of NMR data of plasma and the 82 sets of NMR data of CSF were each pre-processed as described in the “Materials and methods” section. Examples of plasma and CSF spectra are shown in Fig. 2. It is apparent the intensities of many metabolites (normalized to the TSP signal, for visualization purposes only), for example alanine and arginine, are higher in the plasma spectrum than in that from the CSF. Most metabolites present in CSF can be observed in plasma. A few volatile metabolites are not visible in plasma, because of lyophilisation. Some metabolites, for example glutamate and phenylalanine, are detected in plasma only. This is mostly because of the low concentration of these metabolites in CSF. The NMR spectra of CSF were divided into 409 bins, which contain resonances of 33 identified metabolites and some unidentified signals. For plasma, the NMR spectra were divided into 478 bins, which correspond to resonances of 50 identified metabolites and some unidentified signals.

**Fig. 3:**
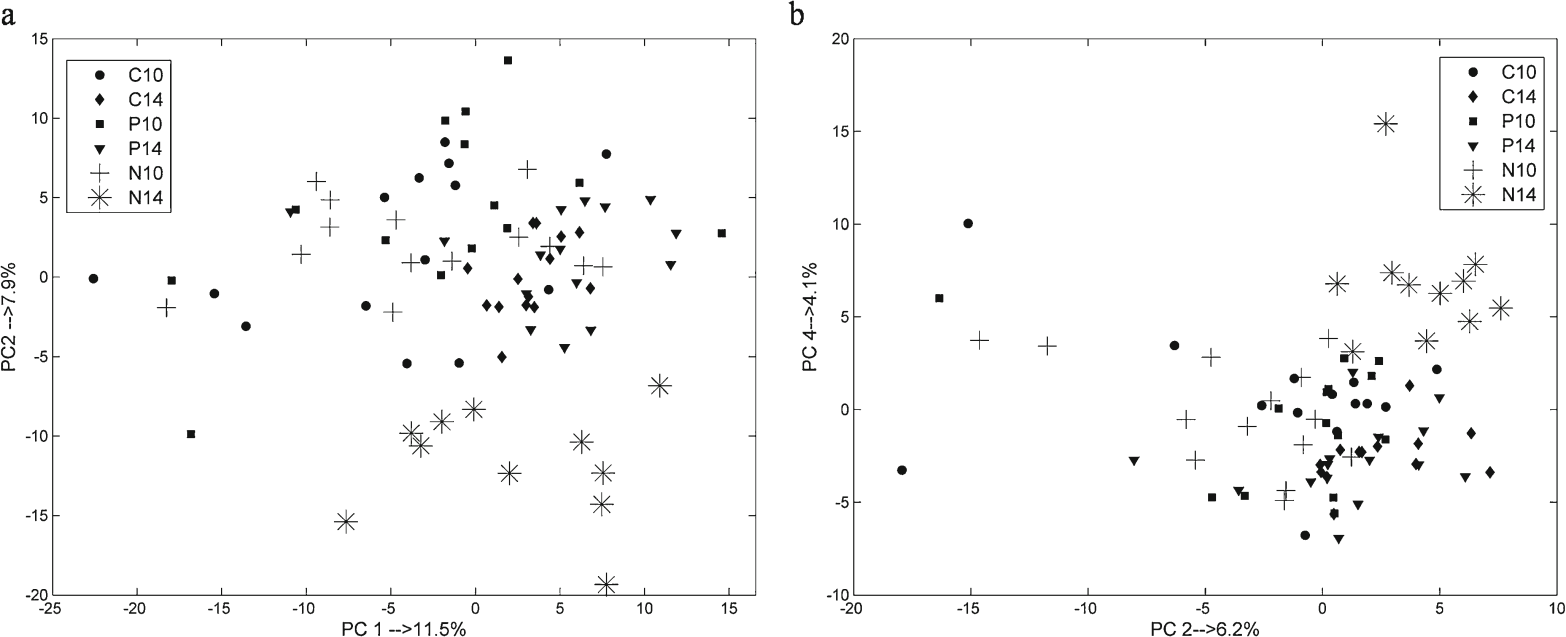
PCA score plot of: **a** plasma NMR spectra; **b** CSF NMR spectra

After pre-processing, explorative analysis was performed by means of R-PCA and PCA. Initially, R-PCA was applied to the autoscaled spectra of 82 rat CSF and plasma samples, to check for outliers. No outliers were detected. Figure 3a and b show the PCA score plots of the plasma and CSF NMR spectra, respectively. These figures show that samples belonging to group “N14” are clearly separated from the other samples along PC2 for plasma data and along PC4 for CSF data. In both situations PC1, which is the main source of variance, does not show any group information. This indicates that a large source of the variance in the data does not correspond to the available groups. No clear grouping is present because most of the groups overlap. It is important to mention that further PCs did not show groupings either.

### Supervised analysis

The most straightforward approach for separating the six groups present in CSF and plasma data simultaneously is to apply a multi-class method, for example PLS2-DA. The two datasets can be analyzed separately by PLS2-DA. Alternatively, the CSF and plasma data can be fused and PLS2-DA can be applied to the fused data. However, PLS2-DA has to describe all group-related variations at the same time. This might lead, on the one hand, to worse results in comparison with multiple binary PLS-DA models and on the other hand, to difficulties in biological interpretation. One can apply binary PLS-DA models to handle individual biological samples (CSF and plasma) and the (mid-level) fused data sets. This implies that for a full description many binary PLS-DA models have to be constructed and optimized. Therefore, we propose and present a new approach, namely HMF. In HMF, a limited number of multiple binary PLS-DA models are used to still fully describe the fused CSF and plasma data. The fusion was achieved by using the approach described in the “Materials and methods” section (subsections “SVM-RFE” and “Classification of individual and fused plasma and CSF datasets”). Binary PLS-DA models were applied to the fused datasets to extract information about the metabolic effects of the different group treatments shown in Table 1 and to establish the significance of the variables. All optimum binary PLS-DA models were constructed using 1LV only. Next these optimized binary PLS-DA models are used in HMF. Below, we first present the results of PLS2-DA, then the binary PLS-DA models, and finally those of HMF. The outcomes of HMF are compared with PLS2-DA of the fused datasets.

#### PLS2-DA—complete EAE model for plasma data, CSF data and fused sets

We applied PLS2-DA to separate simultaneously all six groups of the CSF dataset and of the plasma dataset. The variables included in the PLS2-DA model are selected by linear SVM-RFE. The number of LVs in the PLS2-DA model was optimized by cross-validation. Correct classification for an independent test set was 57.1% for the plasma data and 56% for the CSF (correct classification per class is included in Table S1 in the Electronic Supplementary Material). It is interesting to note that better results are obtained for the binary PLS-DA models (at least for the binary PLS-DA models considered) than for PLS2-DA. However, it is important to mention that PLS2-DA has a more difficult problem to solve (i.e. separate six classes at once) than PLS-DA.

A similar situation is encountered for fused datasets. Correct classification for an independent test set is 65% for PLS2-DA (correct classification per class can be found in the Electronic Supplementary Material, Table S1). This result is much worse than for multiple PLS-DA models. PLS2-DA performance (64% classification) is in turn still much better than a random classifier (correct classification 17%) but still insufficient for proper diagnosis. However, one should notice that some groups are classified completely correctly (100%, e.g. “C10”), whereas others are totally misclassified.

#### PLS-DA models for plasma data, CSF data, and for mid-level fused sets: the onset of neuroinflammation

**Fig. 4:**
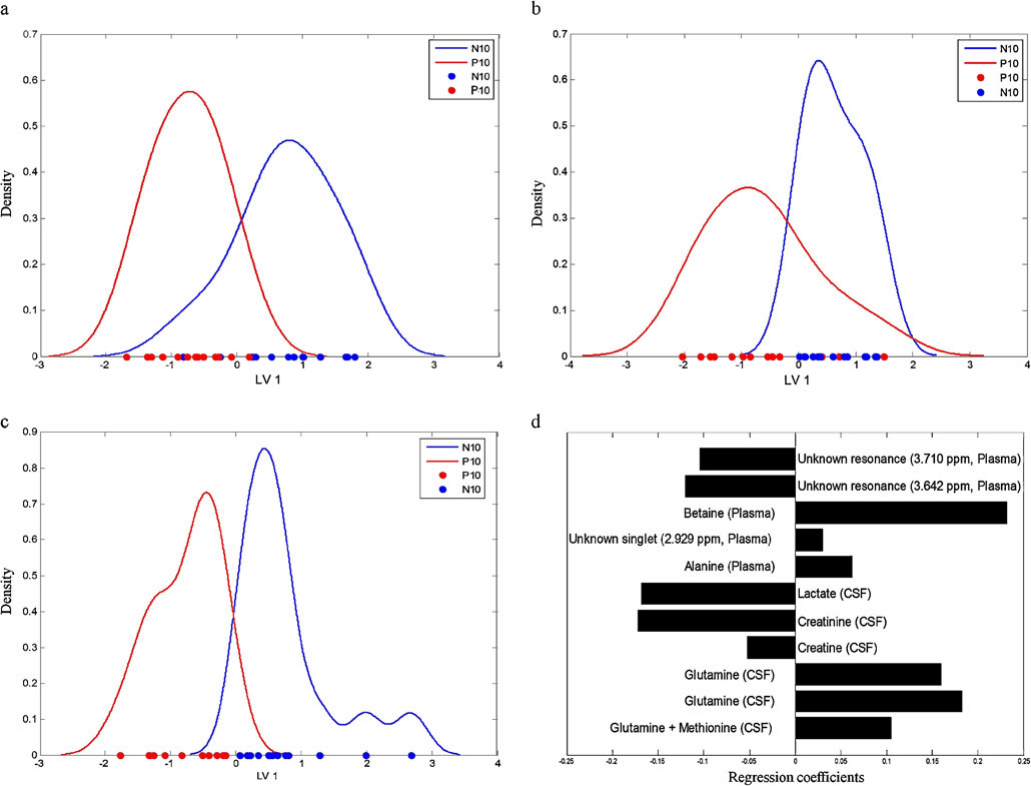
Density distribution of PLS-DA scores of: **a** “P10” vs. “N10” for CSF data; the amount of *y* variance for 1LV is equal to 77.5%; **b** “P10” vs. “N10” for plasma data; the amount of *y* variance for 1LV is equal to 63.5%; **c** “P10” vs. “N10” for fused data; the amount of *y* variance for 1LV is equal to 61.3%; **d** Regression coefficients for fused PLS-DA model

We present the results of PLS-DA obtained for the group “P10” versus “N10”, because this represents the interesting case of early onset of neuroinflammation. Binary PLS-DA models were derived for the separate CSF and plasma data sets and for mid-level fused data sets. The predictive models for the problem “P10” vs. “N10” are displayed as PLS-DA score plots in Fig. 4a, b, and c. These 1LV score plots are presented as the density distribution of the entire group. The PLS-DA model of CSF is constructed on the basis of 87 variables. The PLS-DA model of CSF alone has no prediction ability, as follows from the 50% correct classification for an independent test set. Accordingly the groups “P10” and “N10” (for CSF) are not separated in Fig. 4a. For plasma, the PLS-DA model separates the classes somewhat better, as follows from the classification for independent test sets of 75%. However, there is still quite some overlap and the groups of points are still mixed, as can be seen on the horizontal axis of Fig. 4b.

Because the individual analysis did not furnish satisfactory results, we decided to fuse the selected variables from plasma and CSF data. The SVM-RFE conducted on the fused sets, led to 11 variables (of the 112 initially selected variables). The resulting PLS-DA model of fused datasets is shown in Fig. 4c. Correct classification for the independent test set is 100%, demonstrating the statistical adequacy of this model. As can be seen from Fig. 4c, there is clear separation. Figure 4d shows the regression coefficients of this PLS-DA model. Interestingly, the fusion model consists of six CSF and five plasma variables. This suggests that both biofluids contribute significantly to the group separation.

Table 2 summarizes the results of the PLS-DA models for “P10” versus “N10”, and for two other pairs of groups, namely “C10” vs. “P10”, and “N10” vs. “N14”. These models were used in the HMF. Table 2 lists, with the degree of correct classification for independent test sets, the number of variables selected by RFE-SVM used in PLS-DA models for the fused data. We find that the binary PLS-DA model for “P10 vs. N10” (early onset of neuroinflammation) results in 100% correct classification. The same is true for “N10” vs. “N14” (progression of neuroinflammation), whereas for “C10” vs. “P10” 93% correct classification is achieved. The score plots and regression coefficients of models “C10” vs. “P10” and “N10” vs. “N14” are shown in Figs. S5a–S5d in the Electronic Supplementary Material.

**Table 2:** Correct classification for an independent test set obtained for fused datasets, number of variables from plasma and CSF used in a PLS-DA model, and number of samples in training set and test set

PLS-DA model	Correct classification	No. of variables in PLS-DA model		No. of samples	
		Plasma	CSF	Training	Test
C10 vs. P10	93%	4	13	20	8
P10 vs. N10	100%	5	6	20	8
N10 vs. N14	100%	8	3	18	7

In Table 2 only a few of many possible pairs of groups for PLS-DA have been presented. Nevertheless, in Table S2 in the Electronic Supplementary Material correct classification for the independent test set obtained for individual analysis of plasma data, CSF data, and fused datasets by PLS-DA for different pairs of groups can be found. To achieve full or nearly full description of the fused data set, without having to use all pairs of groups, we apply hierarchical data models fusion, HMF, in the next section.

#### Hierarchical models fusion

**Fig. 5:**
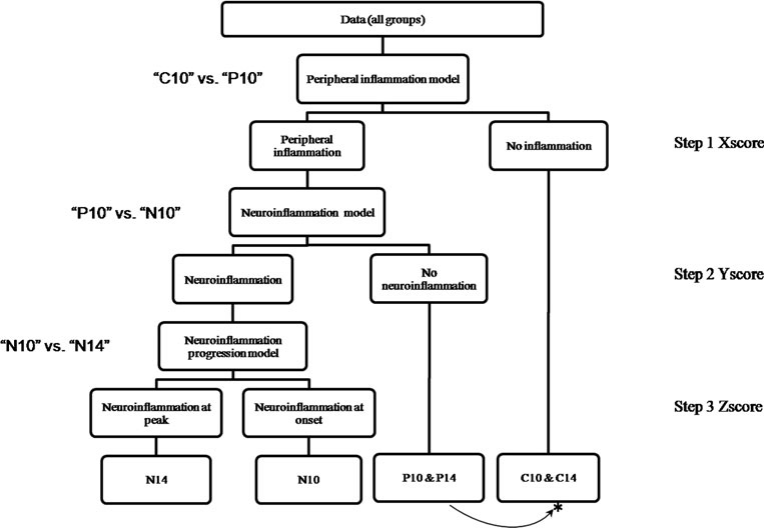
Representation of hierarchical models fusion for fused plasma and CSF NMR datasets. *Asterisk*, note that “P14” is classified as control (the inflammation has gone, see section below)

The predictive power of the individual PLS-DA models is, by itself, already satisfactory (as is apparent from Table 2 and Table S2 in the Electronic Supplementary Material). At this point one could stop the analysis and start biological interpretation of the results. However each PLS-DA model only looks at two groups at a time and is, therefore, not able to predict the results for a completely unknown sample. Thus, it is necessary to combine the different models. The idea of HMF is to join them in a meaningful order. To perform HMF on the CSF and plasma datasets, we used the PLS-DA models of “C10” vs. “P10”, “P10” vs. “N10”, and “N10” vs. “N14” (Table 2). These characterize, respectively, the effect of peripheral inflammation, neuroinflammation, and progress of neuroinflammation. They are, therefore, consistent with the experimental design shown in Table 1. The HMF approach used in this paper is represented in Fig. 5. Note that we present here HMF on the fusion of two datasets but the same principle could be applied to a single dataset. As explained in the “Materials and methods section”, HMF is validated in two ways. First, all individual PLS-DA models were statistically validated with independent test sets. Second, the complete scheme of HMF was validated with a set including all test sets used in the binary PLS-DA models from Table 2 and, additionally, some samples belonging to classes “C14” (four samples in test set and ten in training set) and “P14” (five samples in test set and ten in training set). The graphical representation of HMF for training and test set samples is shown in the Electronic Supplementary Material in Fig. S5. It is apparent all test samples are predicted correctly. The permutation test was also performed for all six classes, as an extra check. The *p*-value for 40,000 permutations was equal to 0.0006.

We started with the PLS-DA model of peripheral inflammation, i.e. “C10” vs. “P10” (shown in Fig. 5 as step 1). This enables one to separate healthy objects, i.e. those without any type of inflammation (Table 1) from all those with peripheral inflammation. The latter also includes groups which have undergone neuroinflammation, because, in accordance with the experimental design shown in Table 1, these groups were injected with CFA. This step enables creation of a first new score (i.e. Xscore) for all samples in the data. In other words, this model separates the healthy groups from those with any form of inflammation (neuro or peripheral).

In a second step, we used a PLS-DA model of “P10” vs. “N10”, shown in Fig. 4d. This model distinguishes peripheral inflammation from neuroinflammation at the onset of EAE. Therefore, by using this model we are able to separate neuroinflamed animals from animals that were only peripherally inflamed (i.e. “P10” and “P14”; shown in Fig. 5 as step 2). Similar to step 1, a second score is generated, the Yscore. At this level, we have separated samples belonging to groups with peripheral inflammation (i.e. “P10” and “P14”) from the neuroinflamed groups (“N10” and “N14”).

**Fig. 6:**
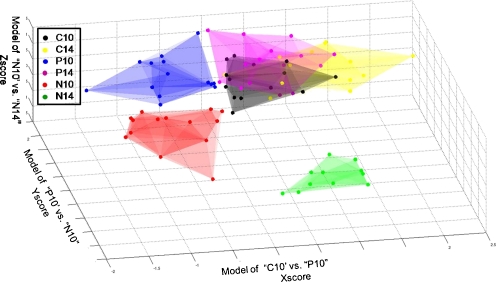
Graphical representation of HMF applied to fused data from plasma and CSF

The last step (number 3) considers the separation of the onset of the disease from the peak of EAE. To achieve this separation, we applied the PLS-DA model of “N10” vs. “N14”, i.e. the model describing the severity of neuroinflammation. At this level, a third new score is created, the Zscore. After iterative application of these simple 1LV models to fused plasma and CSF datasets, we can integrate the three new scores, i.e. Xscore, Yscore, and Zscore. They are then used to visualize the outcome. They represent the relationship between the groups and their separation. The corresponding graph is shown in Fig. 6. As can be observed, full separation of the different groups is achieved. It is worth mentioning that samples belonging to healthy groups “C10” and “C14” mostly overlap. However, a small shift along the *x*-axis is observable, probably because of sampling time (Day 10 vs. Day 14). As can be noticed, samples belonging to group “P14” overlap with healthy groups, which is in agreement with our previous finding that peripheral inflammation has vanished by day 14 [11].

## Discussion

By using a mid-level fusion architecture we were able to identify a set of metabolites that revealed significant changes in the plasma and CSF of neuroinflamed animals. Based on the regression coefficients of the PLS-DA model of fused datasets (for example Fig. 4d and Electronic supplementary material) the importance of the individual metabolites in each PLS-DA model and a direction of elevation/reduction of concentration can be evaluated. On the basis of this information, biological interpretation of these metabolites and their connection to EAE, neural inflammation, and/or MScl can be performed. Therefore, the first aspect to be discussed is the nature of the selected metabolites. It should be mentioned that the main objective of this paper is not to provide a biological explanation, but to present the methodology for fusion and analysis of ^1^H NMR metabolomics datasets. Therefore biological conclusions are not stressed.

One can notice that many (mostly neutral) amino acids were found to be discriminatory for EAE groups and, therefore, we focus on these. Transport of neutral amino acids through the BBB is significant for overall regulation of cerebral metabolism and neurotransmitter production [27]. BBB amino acid transport is important in regulation of several pathways of brain amino acid metabolism. It is known that EAE affects the BBB. It causes disruption of the BBB and affects the saturable transport system of substances involved in the disease process [28]. Injection of CFA can itself lead to increased BBB permeability to small molecules and even specific serum proteins [29].

We found that tyrosine concentration is reduced in plasma of groups “P10”, “N10”, and “N14”. It has been reported previously that tyrosine has a role in BBB permeability [30]. In accordance with our results Monaco et al. detected a reduced level of plasma tyrosine in MScl [31]. Another neutral amino acid related to EAE groups is alanine. This metabolite was found as a relevant metabolite in both plasma and CSF. Its concentration is reduced in the CSF and plasma of EAE groups in comparison with healthy controls and peripheral inflamed group “P10”. Alanine is associated with energy metabolism and is known to be used as a source for pyruvate for energy metabolism and for macromolecules within neural and immune cells [11]. Similarly, lysine concentration was elevated in CSF and plasma from neuroinflamed groups “N10” and “N14”. Qureshi and co-workers in a study on the role of neurotransmitter amino acids in CSF of MScl patients reported increased levels of lysine in CSF and plasma of MScl patients [32].

We found the combination of glutamate and proline signals in plasma decreased in the EAE groups compared with the other groups. In a previous study a change of glutamate concentration in CSF was reported in a clinical study of MScl [33]. Glutamate is a very important neurotransmitter and the most abundant free amino acid in the brain. A metabolite closely interconnected with glutamate is glutamine. This metabolite was found in plasma as discriminatory for groups injected with immune booster (i.e., “P10” and “N10”) when compared with the healthy groups, and its concentration was elevated in these groups. It was also found as discriminatory when comparing “P10” and “N10” groups. In CSF, its level was found to be down regulated in group “P10” in comparison with healthy controls. This metabolite is involved in energy metabolism. It has been shown that glutamine is a necessary nutrient for cell proliferation, serving as a specific fuel for inflammatory cells and enterocytes and, when present in appropriate concentrations, enhancing cell function [34]. The last amino acid that is discussed here is phenylalanine. This metabolite, which was diminished in EAE groups, is the precursor to tyrosine and is needed for function of the catecholamine neurotransmitters epinephrine, norepinephrine, dopamine, and tyramine. In a previous study by Monoco et al. a reduced level of phenylalanine was found in MScl [31].

One aspect which has not been emphasized is the importance of a proper preprocessing. Here the use of AI binning ensures that one bin corresponds to one peak, thus preventing signals from different metabolites being mixed within one bin. Normalization is the second important preprocessing aspect. We compared the effect of classical total area normalization with that of probabilistic quantum normalization. No strong differences were observed (data not shown) therefore we decided to use the simplest approach. However one should be aware that total area normalization could be suboptimum, because of the large effect of highly abundant multiplets (e.g. glucose).

The third aspect to be discussed is connected with the data analysis strategy used in this manuscript, i.e. mid-level data fusion and HMF. First, the approach of mid-level data fusion performed here enabled individual variable selection and thus discard of irrelevant information. Second, the HMF method, shown in this paper, is a novel, simple strategy for multi-class analysis. Each PLS-DA model only looks at two groups at a time and, therefore, a single model cannot predict a completely unknown sample. This is a minor advantage of HMF over multiple PLS-DA models. One should keep in mind that the outputs of this method are statistically accurate, because they are based on validated binary predictive models. Moreover, the complete scheme of HMF was also validated. The output of HMF (i.e. new scores) can be used for visualization or prediction of new samples. However, it is good practice to check if these new scores are orthogonal.

When comparing HMF and PLS2-DA, it is important to mention that it is possible that if some groups do not behave in accordance with the experimental design, the optimum solution for class separation can be flipped. In other words, if one or more groups cannot be distinguished, PLS2-DA still tries to separate them, which may affect the solution for the whole PLS2-DA model. In the case of EAE datasets, there are two groups (i.e. “N10” and “P14”) that are characterized by behaviour different than was assumed by experimental design. For the “N10” group we have previously shown that animals are heterogeneous regarding disease response [11]. Further, the second group “P14” was not (or no longer) peripherally inflamed on day 14. This causes the results obtained by PLS2-DA to be sub-optimum for groups “N10”, “C14”, and “P14”. In the method proposed here, HMF, the situation described for PLS2-DA cannot happen. HMF leads to the optimum solution, because it includes relevant sources of variance between groups individually rather than all at the same time. This suggests that if two groups are not separable this can be easily detected during the HMF and does not affect the separation between other groups.

In our study the HMF was shown for fused plasma and CSF NMR datasets. However this approach can be also used for one type of sample (as shown in the Electronic supplementary material). Obviously, the individual PLS-DA models can be developed for any type of sample and then HMF can be applied.

## Conclusions

In this study we have demonstrated the feasibility of fusion of metabolomics ^1^H NMR datasets from different biofluids. From the perspective of data analysis multiple challenges had to be addressed. One was concerned with the biological variation usually encountered in omics experiments. Another issue was linked to the number of variables recorded by NMR, which is, first, much greater than to the number of samples and, second, most are probably unrelated to the studied problem or redundant. We successfully solved these problems using a new architecture for data fusion in which SVM-RFE is used as variable selection method and PLS-DA to focus on the information of interest through a training procedure.

We analyzed CSF and plasma metabolomics data of the EAE model for MScl using mid-level data fusion. The procedure was represented by constructing a predictive model for neuroinflamed group “N10”, i.e. before physical symptoms have appeared, versus a peripherally inflamed group “P10”. Prediction models based on either CSF or plasma metabolomics data alone could not separate the immune booster and EAE groups at day 10, whereas the predictive model using a fused set of variables from CSF and plasma managed to separate the two groups with 100% correct classification for the independent test set. One should be aware that these results do not imply that all new samples will be always correctly classified. However validation with the independent test set and the permutation test set indicates the results are meaningful. This shows that by using bio-molecular information (metabolomic data), a diagnosis can be made before physical symptoms arise. Our results also demonstrate that plasma can be of significant importance in the diagnosis of neuroinflammation. Therefore, we believe that plasma should be considered when investigating neuroinflammation.

Finally, we have introduced a new multi-class method, HMF, which describes relevant sources of variance connected with groups’ description by fusing individual binary models. We have shown that by using HMF we are able to separate groups in our data by using simple, easily interpretable, one-component predictive models.

From a biological perspective, the selected metabolites seem to be relevant, because the metabolites described in this study were previously found to be related to EAE and/or MScl. Therefore, they provide biological validation for the fusion of data from two different biofluids.

Further research will focus on deeper interpretation and absolute quantification of newly detected metabolites in plasma and CSF and their relationship to BBB. These two steps are time-consuming but would give more insight into the mechanism of the disease. The pattern and concentrations defined by these variables could also be studied by themselves and put into a systems biology context. Absolute quantification would be crucial for obtaining advanced biological conclusions and conformation using a completely different analytical method (e.g. mass spectrometry).

## Electronic supplementary material

Below is the link to the electronic supplementary material.

ESM 1 (PDF 586 kb)
