# Supplementary material for: Simultaneous analysis of plasma and CSF by NMR and hierarchical models fusion
Source: Anal Bioanal Chem. 2012 Mar 7;403(4):947–59. doi: 10.1007/s00216-012-5871-4 (PMC3336062; doi:10.1007/s00216-012-5871-4)

Analytical and Bioanalytical Chemistry

Electronic Supplementary Material

Simultaneous analysis of plasma and CSF by NMR and hierarchical models fusion

Agnieszka Smolinska, Joram Posma, Lionel Blanchet, Kirsten A.M. Ampt, Amos Attali, Tinka Tuinstra, Theo Luider, Marek Doskocz, Paul J. Michiels, Frederic C. Girard, Lutgarde M.C. Buydens, and Sybren S. Wijmenga

Figure S1. Typical disease progression in EAE model.

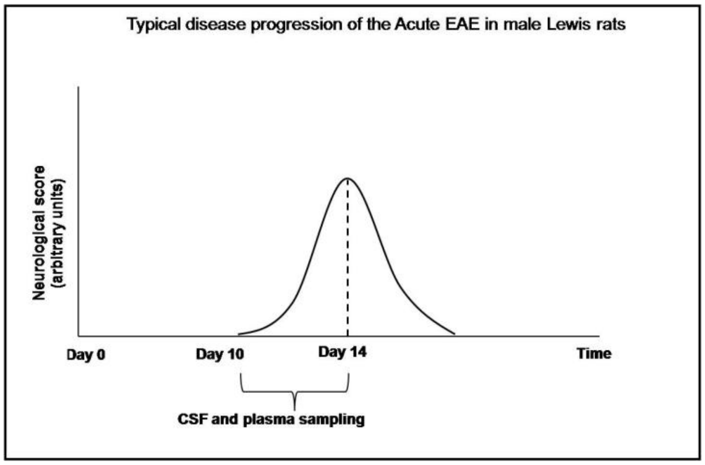

<u>Simulated data</u>

In order to represent the use of Hierarchical Model Fusion (HMF) a simulated data set was constructed. This data set contains 280 samples (corresponding to four classes, i.e. 70 samples per class) and 105 variables. It was simulated in such way that the samples are represented by random vectors chosen from multivariate normal distribution. 5 variables are informative and 100 are irrelevant for the discriminating classes. The informative traits were incorporated with random noise (approximately 6% of informative signal). The data were simulated in such way that: classes 1 and 2 overlap with each other as well as classes 3 and 4, while classes 2, 3 and 4 are the most dissimilar one. In Figure S2 the scatters plot of the simulated data in the plane of variable 5 and 2 (both informative) and Principal Component Analysis (PCA) score plot are shown. As can be observed from Figure S2a these two variables allow for discriminating classes. On the contrary, the PCA score plot (of autoscaled data, see Figure S2b) does not reveal any groupings. Indeed, the four classes completely overlap in the plane defined by PC1 and PC2.

Figure S2. Visualization of simulated data: (a) the scatter plot in the plane defined by the informative variables 5 and 2; (b) PCA score plot in the plane of PC1 and PC2.

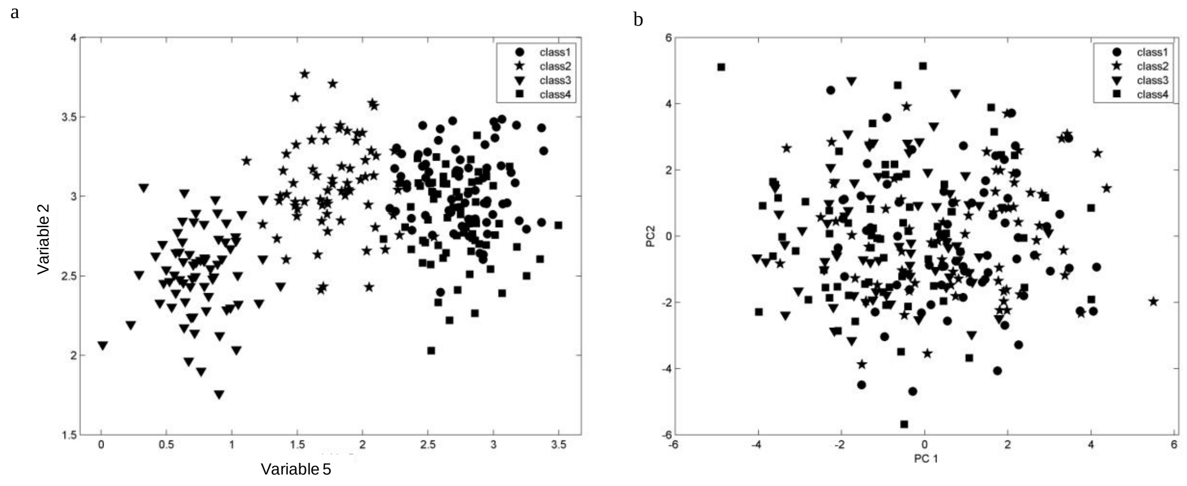

The data were divided into model (52 samples per class) and independent test set (18 samples per class) using Duplex algorithm. In order to perform HMF three PLS-DA models were constructed, namely class 1 vs. class 2, class 2 vs. class 3 and class 3 vs. class 4 for autoscaled data. They were selected based on the information how the classes were simulated. The correct classification rate for the independent test set was equal 94.4%, 100% and 94.4%, respectively for the PLS-DA models.

These three PLS-DA models were next used to obtain three new scores following the HMF procedure. We started with PLS-DA model of class 1 vs. class 2. This step enables of creating Xscore. This allows one to separate class 1 from the rest. In the next step PLS-DA model of class 2 vs. class 3 was used. Similarly to the first step a second score is generated, i.e. Yscore. At this point class 2 is separated. In the final step PLS-DA model of class 3 vs. 4 was utilized leading to Zscore. By concatenating these three new scores a graphical representation is obtained, showing all classes at once. In Figure S3 the graphical representation of the HMF applied to simulated data is presented. Note that this figure shows where the test samples (18 samples per class) are projected into these new scores. As can be noticed the test samples are correctly projected (95.3% test samples are correctly predicted by HMF). This demonstrates the

statistical relevance of the obtained results. To show if classification results are better than any other random classification a permutation test was made. Of 3000 runs none had correct classification bigger than 95.03%, leading to p-value 0.0003.

To evaluate the performance of HMF we constructed PLS2-DA model on the same training set used for HMF. The correct classification for the independent test set was equal to 83.3%. This result shows that PLS2-DA underperforms compared to HMF.

Figure S3. Graphical representation of the results of the HMF obtained on the simulated data.

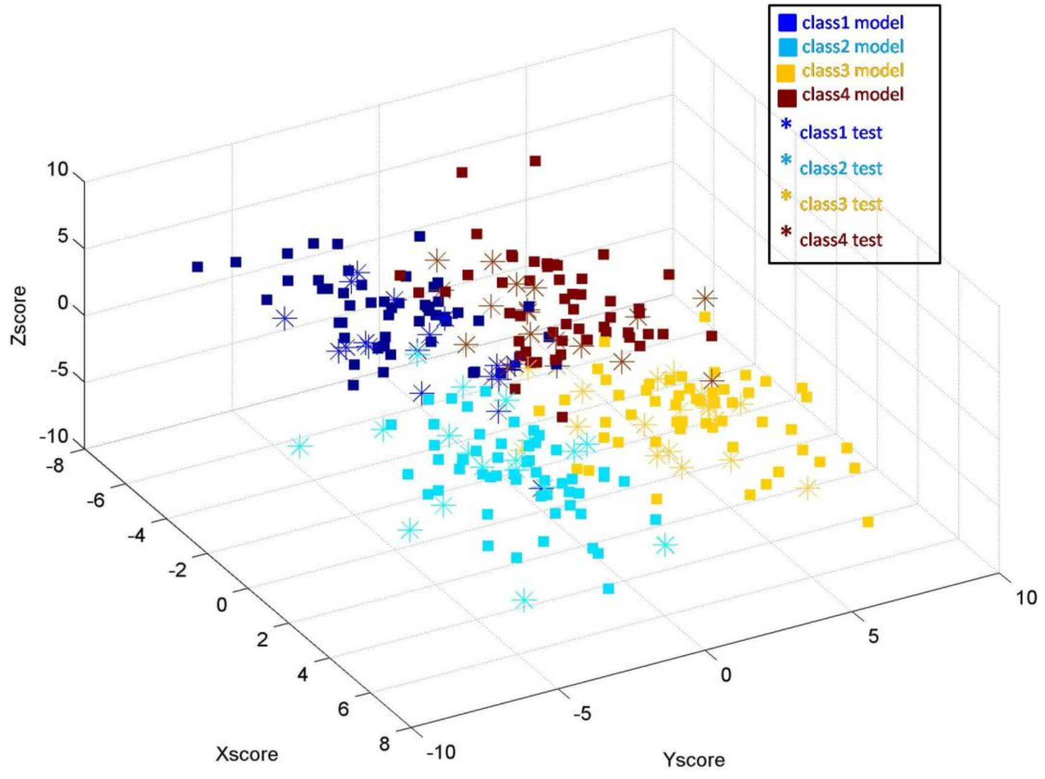

Addition of different noise levels

In order to represent the effect of noise we added different levels of homoscedastic and heteroscedastic noise to informative variables in the simulated data. We created 6 different levels and we repeated it 60 times. Every noise level comprised different percentage of informative traits (see Figure S4 on x-axis). 6 noise levels and 60 repetition leads to 360 runs. For each run PLS-DA models were recalculated and HMF was applied as described above. Moreover we performed permutation test (3000 runs) to reduce the possibility of random classification.

In Figure S4 the average correct classification and standard deviation obtained for HMF applied to simulated data with different levels of noise in informative traits is presented. As can be observed the higher the noise level the less accurate results have become. The algorithm performs well in terms of correct predictions for independent test set up to situation number 4 (i.e. data containing 35% of noise in comparison to informative traits). However, for data containing 35% of noise the results become unstable, since the outcome of permutation test shows that for 3000 permutations some had higher correct classification than for the original classification. For the first three noise levels, of the 3000 permutations none had a number of correct classifications higher than for original classification, leading to a p-value of 0.0003.

Figure S4. Average correct classification rate and standard deviation obtained for simulated data with 6 different noise levels. In the x-axis amount of noise with respect to relevant variables is indicated. For each noise level the results from permutation test are included on top of the triangular.

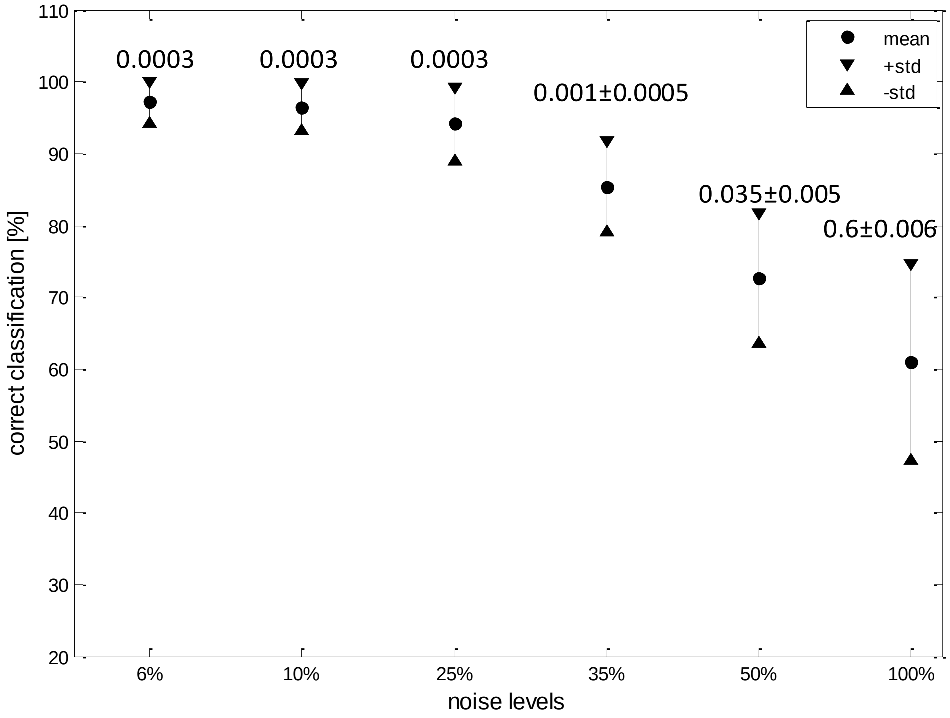

Pseudo code for LOO CV used for variable selection and model optimization

Assuming a data matrix **X** for training set of size (n x p, where n is a number of samples and p a number of variables) a LOO CV is performed (separately for plasma and CSF) according to the following scheme, which was repeated for each sample (i) in **X**:

Beginning of LOO CV

*1. One object (i) is removed from training data matrix **X***

*2. Autoscaling of the data matrix **X** with remaining objects (of size m-1 x p)*

*3. RFE is performed on the autoscaled data matrix **X** with remaining objects (of size m-1 x p)*
4. A ranking of variables is obtained
End of LOO CV

In next step a final ranking for variables is obtained.
CSF and plasma training sets are concatenated and variables ranking is again performed with LOO CV; note that here **X** corresponds to concatenated plasma and CSF data:

Beginning of LOO CV
*1. One object (i) is removed from training data matrix **X***
*2. Autoscaling of the data matrix **X** with remaining objects (of size m-1 x p)*
*3. RFE is performed on the autoscaled data matrix **X** with remaining objects (of size m-1 x p)*
4. A ranking of variables is obtained
End of LOO CV

The final ranking for concatenated data is obtained.

Optimization of PLS-DA model complexity (number of latent variables) for data matrix **X** (repeated for each object (i) in **X**). Note that here **X** can correspond to CSF data, plasma data or fused data sets:
Beginning of LOO CV
*1. One object (i) is removed from training data matrix **X** (m x p)*
*2. Autoscaling of the data matrix **X** with remaining objects (of size m-1 x p)*
*3. Autoscaling of removed object (i) with mean and standard deviation delivered from the data matrix **X** with remaining objects*
*4. Fit PLS-DA model to autoscaled data matrix **X** with remaining objects (size m-1 x p)*
5. Classify removed object (i)

6. Calculation of the root mean square error of cross-validation (RMSECV).

End of LOO CV

Model complexity=min(RMSECV)

Figure S5. Graphical representation of the results of the HMF obtained on the fused CSF and plasma datasets with indication of training and test samples.

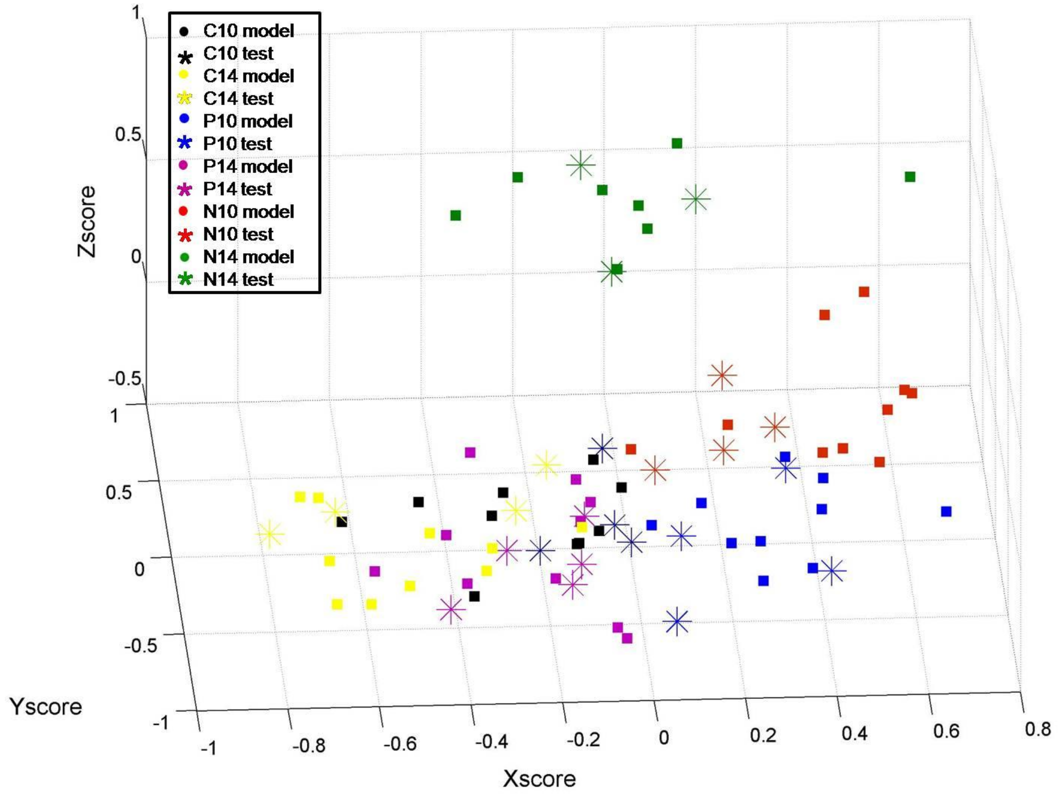

Table S1. Correct classification rate for independent test set obtained for individual analysis of plasma data, CSF data and fused datasets by PLS2-DA. Complexity of PLS2-DA models LV=3. Number of variables: 20 variables for plasma and 20 variables for CSF.

Groups	plasma	CSF	fused (plasma and CSF)
C10	50%	67%	80%
P10	100%	67%	80%
N10	0%	0%	67%
C14	0%	0%	0%
P14	80%	100%	67%
N14	100%	100%	100%

Table S2. Correct classification rate for independent test set obtained for individual analysis of plasma data, CSF data and fused datasets by PLS-DA.

PLS-DA model	plasma	CSF	fused (plasma and CSF)
C10 vs. P10	62.50%	62.50%	93%
P10 vs. N10	75%	50%	100%
C10 vs. N10	62.5%	100%	100%
N10 vs. N14	62.5%	100%	100%
C14 vs. N14	100%	100%	100%
C14 vs. P14	100%	100%	100%

Figure S6. Density distribution of PLS-DA scores of fused data: (a) “C10” vs. “P10”, the amount of y variance for 1 LV is equal 76.2%; (b) Regression coefficients of “C10” vs. “P10” PLS-DA model; (c) “N10” vs. “N14”, the amount of y variance for 1 LV is equal 94.04%; (d) Regression coefficients of “N10” vs. “N14” PLS-DA model.

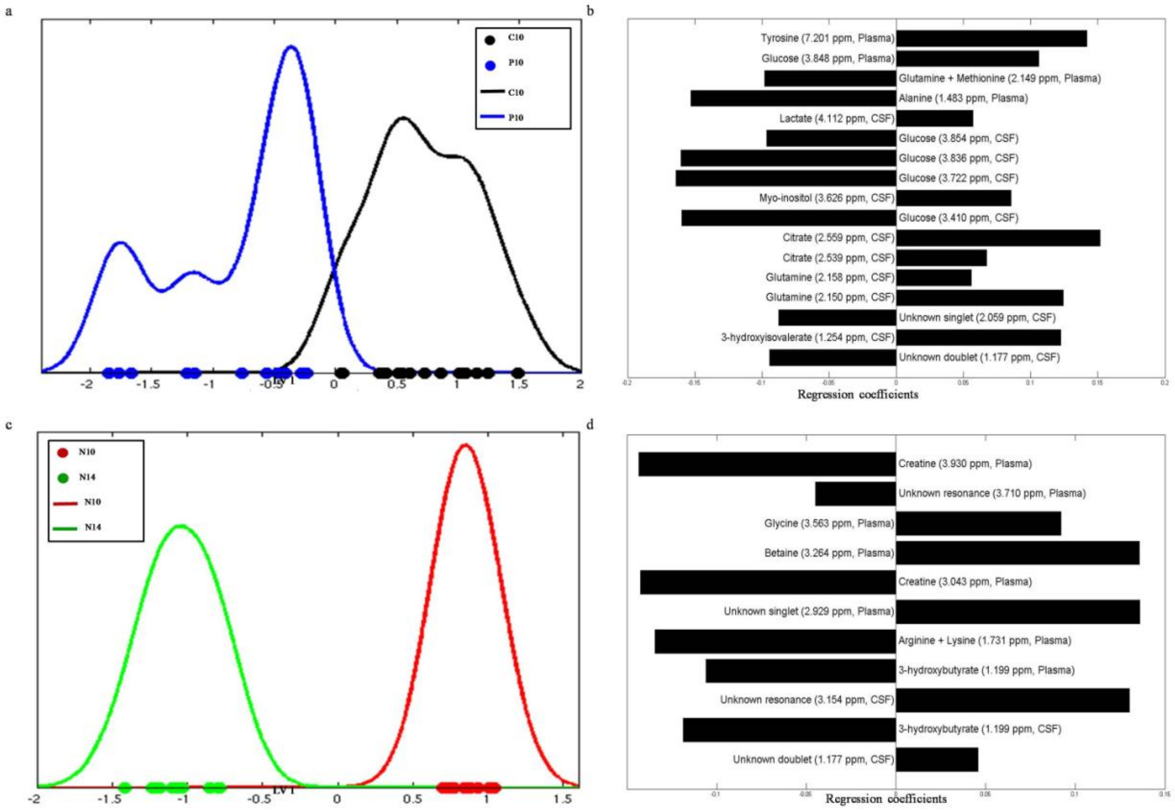

Supplement: Supplementary file 1 — (PDF 586 kb) [file 216_2012_5871_MOESM1_ESM.pdf]
